# Case report and literature review: isolated ACTH deficiency induced by combined nivolumab and trastuzumab therapy in gastric adenocarcinoma

**DOI:** 10.3389/fendo.2026.1742701

**Published:** 2026-04-20

**Authors:** Liwei Shi, Jinrong Wang, Xiaohong Xie, Wei Gu

**Affiliations:** 1 Department of Internal Medicine, Graduate School of Hebei Medical University, Shijiazhuang, China; 2 Department of Endocrinology, Harrison International Peace Hospital, Hengshui, China; 3 Department of Critical Care Medicine, Harrison International Peace Hospital, Hengshui, China

**Keywords:** adrenocorticotropic hormone deficiency, dumping syndrome, immune-related adverse events, nivolumab, trastuzumab

## Abstract

**Background:**

Isolated adrenocorticotropic hormone (ACTH) deficiency (IAD) is an uncommon endocrine disorder characterised by the selective reduction or absence of pituitary ACTH secretion, resulting in secondary adrenal insufficiency while other pituitary hormone axes remain functional. The clinical manifestations of this condition are frequently non-specific, and may include symptoms such as fatigue, decreased appetite, weight loss, hypotension, and hyponatremia. These symptoms can be easily confused with those of other diseases, resulting in either missed diagnoses or misdiagnoses. Glucocorticoid replacement therapy is generally considered to provide effective symptom relief and a favourable prognosis. In recent years, the widespread use of immune checkpoint inhibitors (ICIs) in cancer treatment has resulted in a significant increase in immune-related adverse events (irAEs). Isolated ACTH deficiency has been reported as a rare yet severe endocrine-type irAE, potentially arising from immune-mediated pituitary injury. In view of the nonspecific nature of its clinical presentation, early recognition and timely intervention are of critical importance.

**Case presentation:**

The present study reports the case of a patient diagnosed with gastric adenocarcinoma who underwent laparoscopic total gastrectomy, followed by combination therapy with oxaliplatin (OXA), nivolumab, and trastuzumab. Approximately eight months into the treatment regimen, the patient exhibited symptoms of isolated ACTH deficiency and hypothyroidism, indicative of a multi-endocrine system adverse reaction. Such multi-system endocrine dysfunction following this combination therapy is relatively uncommon. The present study analyses the patient’s clinical presentation, laboratory findings, and imaging data, and discusses the differential diagnosis from post-gastrectomy dumping syndrome.

**Conclusion:**

This case demonstrates that in patients with malignant tumours receiving nivolumab and trastuzumab therapy, the presence of unexplained hypoglycaemia, persistent fatigue, anorexia, nausea, or vomiting – non-specific symptoms – should raise high suspicion for immune-related endocrine adverse reactions. It is imperative to emphasise the significance of preliminary testing for serum cortisol, ACTH, and thyroid function levels. Early recognition and prompt initiation of hormone replacement therapy can effectively prevent misdiagnosis, missed diagnosis, and delayed treatment.

## Introduction

The widespread application of immune checkpoint inhibitors (ICIs) in the treatment of various malignancies has led to a growing focus on immune-related adverse events (irAEs). In addition to the well-documented toxicities affecting the skin, gastrointestinal system and lungs, there is an increasing body of evidence pointing to the potential for endocrine system damage. Among these, isolated adrenocorticotropic hormone (ACTH) deficiency (IAD), though rare, can lead to severe secondary adrenal insufficiency. It is imperative to recognize the signs and symptoms of the condition in question, and to act promptly in order to avoid life-threatening consequences.

The aetiology of IAD remains incompletely understood, with potential triggers including head trauma, lymphocytic hypophysitis, autoimmune hypophysitis (often coexisting with other autoimmune diseases), certain medications, and radiation therapy for intracranial tumours (1). In 1954, Steinberg et al. reported the first case of early-onset isolated ACTH deficiency (2). Advances in diagnostic techniques and endocrine function testing have resulted in a gradual increase in case reports, although the condition remains rare overall. The recent emergence of IAD cases associated with immunotherapy suggests that ICIs may impair pituitary function through immune-mediated mechanisms, thereby triggering this disorder. The current state of literature on IAD occurring after immunotherapy in gastric cancer patients is extremely limited, with cases following combined nivolumab and trastuzumab treatment being particularly rare. The clinical manifestations of this condition are often non-specific, and can easily be confused with postoperative complications (such as dumping syndrome) or adverse reactions to chemotherapy. This can result in a delay in diagnosis and treatment.

This study reports a case of isolated ACTH deficiency with hypothyroidism and multiple endocrine system involvement occurring approximately eight months after combination therapy with oxaliplatin (OXA), nivolumab, and trastuzumab in a patient with gastric adenocarcinoma following laparoscopic total gastrectomy. A comprehensive analysis of the clinical course, endocrine characteristics, and relevant literature is presented in this case study, with the aim of exploring the potential pathogenesis, diagnostic criteria, and clinical differential considerations of immune therapy-associated IAD. This approach is intended to enhance clinicians’ ability to recognize and manage this rare immune-related endocrine adverse reaction.

## Case summary

The patient is a 45-year-old female who was admitted on 28 October 2023. Her weight is 43 kg and she presented with a history of “poor feeding for 9 months post-gastric cancer surgery and confusion for 3 hours”. A gastroscopy was performed on the patient during a routine physical examination in August 2022. This revealed the presence of asymptomatic gastric cancer, although the specific details of this diagnosis are not available. On 9 March 2023, she underwent laparoscopic total gastrectomy under general anaesthesia. Postoperative pathology confirmed the presence of gastric adenocarcinoma, with no evidence of metastasis on lymph node dissection. Subsequent to the operation, the patient underwent six cycles of adjuvant chemotherapy, consisting of the regimen OXA, trastuzumab and nivolumab. Following the initiation of treatment, a diagnosis of primary hypothyroidism was made, and the patient was prescribed levothyroxine (112.5 μg daily) by mouth. Following the restoration of thyroid function, the patient was discharged from hospital. In the period following hospital discharge, the patient exhibited an inadequate appetite and a diminished nutritional intake. Consequently, they received intravenous nutritional support at a local community hospital. On 26 October 2023, the patient’s condition deteriorated further, with the patient only being able to consume minimal amounts of rice porridge. No symptoms consistent with nausea, vomiting, abdominal pain, diarrhoea, or fever were observed, and no specific intervention was performed. Two days later, the patient exhibited signs of confusion, accompanied by cold sweats, but not limb convulsions or incontinence. The patient was subsequently transferred to a nearby medical facility by emergency medical services. Emergency arterial blood gas analysis revealed the following values: pH 7.34 (reference range: 7.35-7.45), PCO_2_ 43 mmHg (reference range: 35-45mmHg), PO_2_ 116 mmHg (reference range: 80-100mmHg), HCO_3_^-^ 23.2 mmol/L (reference range: 21-27mmol/L), BE -2.5 mmol/L (reference range: -3-3mmol/L), SO_2_ 100.0% (reference range: 95-99%), Na^+^ 123 mmol/L (reference range: 135-145mmol/L), K + 3.4 mmol/L (reference range: 3.5-4.5mmol), Glu 2.0 mmol/L (reference range: 3.3-5.3mmol/L). The immediate administration of 40 ml of a 50% glucose solution intravenously via a slow push gradually improved the patient’s impaired consciousness. The patient was subsequently admitted to the emergency ward for further diagnosis and treatment.

Admission Physical Examination: The patient’s temperature was recorded at 36.6 °C, their pulse rate was 60 beats per minute (bpm), their respiratory rate was 20 breaths per minute (bpm), their blood pressure was 91/71 mmHg, they were alert and oriented, they were malnourished, their abdomen was tender, and there were no other significant findings. The patient’s past medical history is as follows: The patient’s medical history includes a one-year period of “anxiety state”, during which the patient intermittently took “Paroxetine Hydrochloride Tablets 20mg twice daily and Mirtazapine 30mg once daily”. The subject has experienced a weight reduction of 20 kilograms over the past six months. The menstrual history of the patient is as follows: The normal menstrual cycle is characterised by a moderate flow of bright red blood, the absence of clots, normal vaginal discharge and no dysmenorrhea. Pre-treatment laboratory tests: Complete blood count: The WBC count was 2.09×10^9^/L (reference range: 3.5-9.5×10^9^/L). The red blood cell count was 3.72×10^12^/L (reference range: 3.68-5.13×10^12^/L). The haemoglobin concentration was 107 g/L (reference range: 115–150 g/L). The platelet count was 95×10^9^/L(reference range: 100-300×10^9^/L). Biochemical tests: The patient’s sodium level was found to be 124.5 mmol/L (reference range: 137–147 mmol/L), potassium level was 3.73 mmol/L (3.5-5.3 mmol/L), calcium level was 1.92 mmol/L (reference range: 2.11-2.52 mmol/L), calcium ion level was 0.96 mmol/L (reference range: 1.10-1.35 mmol/L), chloride level was 94.6 mmol/L (reference range: 99–110 mmol/L), and liver and kidney function was found to be generally normal. The results indicate that pancreatic function, fasting C-peptide, and β-hydroxybutyrate levels are all within the normal range. Thyroid function: The patient’s free triiodothyronine (FT3) level was found to be 3.300 pmol/L (reference range: 3.53-7.37 pmol/L), while the free thyroxine (FT4) level was 4.480 pmol/L (reference range: 7.98-16.02 pmol/L). Furthermore, the serum thyroid-stimulating hormone (TSH) level was found to be >46.300 mU/L (reference range: 0.56-5.91 mU/L), the total serum triiodothyronine level was 0.850 nmol/L (reference range: 0.92-2.28 pmol/L), and the total serum thyroxine level was 24.190 nmol/L (reference range: 69.71-163.95 pmol/L). These findings were consistent with hypothyroidism. The patient reported discontinuing levothyroxine (Euthyrox) 10 days prior to admission. Following admission, levothyroxine 125 μg was administered once daily, in conjunction with intravenous nutritional support therapy.

During the patient’s period of hospitalisation, blood glucose levels fluctuated between 3.4 and 4.5 mmol/L. Emergency physicians attributed the hypoglycaemia to post-gastrectomy dumping syndrome following total gastrectomy for gastric cancer. The investigation was not given a high priority, and the cause was not confirmed; instead, only caloric supplementation was administered.

During the patient’s period of hospitalisation, symptoms of anxiety were repeatedly observed. The treatment regimen comprised oral administration of mirtazapine tablets and paroxetine hydrochloride tablets. The patient was advised to adopt a positive mindset and a gradual approach to eating, involving the consumption of smaller, more frequent meals, thorough chewing, and a focus on low-carbohydrate, high-protein, fibre-rich foods, with ample fruits and vegetables. However, there was no improvement in symptoms. On 3 November 2023, the patient presented with hand tremors, generalised convulsions, dizziness and fatigue. Consequently, the random blood glucose level was measured at 4.3 mmol/L. Further enquiry revealed that the patient had previously experienced two episodes of generalised convulsions prior to their admission, with no significant differences observed between these episodes and the current presentation. Cranial CT revealed no significant abnormalities.

A consultation with an endocrinologist was requested. On 18 November 2023, the patient was transferred to the endocrinology ward for further testing. This testing included the following: random urine cortisol, ACTH rhythm, and sex hormones. The specific outcomes are delineated in **Table 1**. The analysis of the relevant hormone results indicated the presence of isolated ACTH deficiency. Following the intravenous infusion of 10 mg of hydrocortisone, the patient reported symptoms of restlessness, fatigue, and insomnia. Consequently, the treatment was transitioned to oral prednisolone 5mg. The dosage of prednisolone was meticulously adjusted on the basis of the patient’s clinical condition, culminating in a dosage of 3.75 milligrams in the morning and 1.25 milligrams in the afternoon. Conversely, the blood glucose levels exhibited stability. Subsequent monitoring revealed that electrolyte levels remained within normal parameters, and there was no report of nausea or significant fatigue. The patient’s mental state and appetite were satisfactory, and there was a favourable response to treatment, which resulted in their discharge from hospital. The duration and dosage of hormone use during the patient’s endocrinology department hospitalization, along with blood glucose levels during treatment, are shown in **Table 2**. Following discharge, the patient attended a series of follow-up visits at the outpatient clinic. The specific laboratory test results are displayed in **Table 3**.

**Table 1:** Physiological and biochemical indexes of patient during hospitalization.

Examination dates	October 28, 2023	October 29, 2023	October 31, 2023	November 3, 2023	November 5, 2023	November 6, 2023	November 8, 2023	November 11, 2023	November 14, 2023
K+/(mmol/L)	3.73	4.36		4.38	3.74	4.43	4.34	3.46	3.78
Na+/(mmol/L)	124.5	133.3		135.3	127.6	121.6	123.7	138.9	138.3
Cl-/(mmol/L)	94.6	102.7		104.8	96.2	90.9	94.8	106.5	108.9
ACTH(08:00)/(pg/ml)						0.001			
FT3/(pmol/L)			3.3						3.72
FT4/(pmol/L)			4.48						12.73
TSH/(uIU/mL)			>46.3						5.05
COR(24:00)/(nmol/L)						21.19			
COR(08:00)/(nmol/L)						24.64			
COR(04:00)/(nmol/L)						21.92			
FC-P/(ng/mL)		1.01							
FIns/(uU/mL)		1.96							
LH/(mIU/ML)						2.13			
FSH/(mIU/ML)						6.48			
PRL/(ng/ml)						15.19			
P/(ng/ml)						0.06			
T/(ng/ml)						0.08			
E2/(pg/ml)						49.63			

K+, potassium ion; Na+, sodium ion; Cl-, chloride ion; ACTH, adrenocorticotropic hormone; FT3, free triiodothyronine; FT4, free thyroxine; TSH, thyroid-stimulating hormone; COR, cortisol; FC-P, fasting C-peptide; Flns, fasting insulin; LH, luteinizing hormone; FSH, follicle-stimulating hormone; PRL, prolactin; P, progesterone; T, testosterone; E2, estradiol.

**Table 2:** The duration of hormone use and dosage and blood glucose monitoring during the patient’s hospitalization.

Date	Hormone usage time	Hormone dose (mg)	Blood glucose fluctuation (mmol/L)			
			6 o’clock blood glucose	Blood glucose before dinner	22:00 blood glucose	Pre-meal blood glucose fluctuation
November 8, 2023	14:00	Hydrocortisone 10 mg (intravenous)	/	4.7	5.6	/
November 9, 2023	09:40	Hydrocortisone 10 mg (intravenous)	4.0	6.3	6.1	/
November 10, 2023	08:00	5(Prednisone oral)	4.1	/	/	3.1-4.1
November 11, 2023	08:00	2.5(Prednisone oral)	/	/	/	/
	Afternoon	2.5(Prednisone oral)				
November 12, 2023	08:00	2.5(Prednisone oral)	/	/	/	3.8-7.2
	Afternoon	2.5(Prednisone oral)				
November 13, 2023	08:00	3.75(Prednisone oral)	/	/	/	3.6-4.3
	Afternoon	1.25(Prednisone oral)				
November 14, 2023	08:00	3.75(Prednisone oral)	/	/	/	/
	Afternoon	1.25(Prednisone oral)				

**Table 3:** Patient follow-up status.

Examination dates	December 12, 2023	February 16, 2024	April 22, 2024	May 29, 2024	July 22, 2024	September 18, 2024	January 5, 2025	March 3, 2025	September 12, 2025
K+/(mmol/L)	3.8	3.94	3.92	4.15	4.32	4.22	4.53		4.13
Na+/(mmol/L)	139.2	138.1	141.4	138.6	139.8	136.8	138.9		141.5
Cl-/(mmol/L)	107.3	102.3	104.8	104.8	109.5	103.2	107.4		108.0
ACTH(08:00)/(pg/ml)		0.18		0.213					
FT3/(pmol/L)	3.98	4.23	3.91	4.39	3.22	3.82	3.95	3.81	4.23
FT4/(pmol/L)	14.36	13.13	10.44	9.07	9.91	6.83	9.32	14.7	13.13
TSH/(uIU/mL)	0.069	0.034	0.534	2.946	1.711	19.026	0.893	0.495	0.034
COR(08:00)/(nmol/L)		2.87		5.79				<6.90	
LH/(mIU/ML)		8.00							
FSH/(mIU/ML)		9.17							
PRL/(ng/ml)		9.75							
P/(ng/ml)		0							

## Discussion

The patient presented with a gastric adenocarcinoma and had previously undergone laparoscopic total gastrectomy. On 9th March 2023, the patient received six cycles of combination therapy comprising OXA, nivolumab and trastuzumab. During the treatment period, routine blood counts, coagulation function, and cardiac enzyme panels remained unremarkable. Following the initiation of the treatment plan one month previously, the patient exhibited symptoms consistent with primary hypothyroidism. Consequently, the patient was prescribed levothyroxine supplementation. Consequently, the patient was admitted to our hospital suffering from hypoglycaemia and gastrointestinal reactions. Laboratory tests revealed decreased levels of ACTH and cortisol. In consideration of the patient’s history of targeted therapy utilisation and a comprehensive review of the extant literature, there is a suspicion that the combination of nivolumab and trastuzumab may result in hypothyroidism and isolated ACTH deficiency. Following the administration of treatment, the patient’s condition was stabilised.

Nivolumab is a monoclonal antibody that targets programmed cell death protein-1 (PD-1). The process of blocking the interaction between PD-1 and its ligand, programmed death ligand-1 (PD-L1), has been shown to enhance the body’s antitumour immune response. The drug has been approved for the treatment of multiple cancer types, including non-small cell lung cancer, melanoma, and renal cell carcinoma. PD-L1, thereby enhancing the body’s antitumor immune response (3, 4). The over-expression of PD-L1 is a pivotal mechanism by which certain tumours evade immune system attacks (5, 6). While the efficacy of PD-1 inhibitors in promoting immune system-mediated destruction of tumour cells is well-documented, the disruption of immune checkpoints that results from such treatment can lead to adverse reactions by disturbing the delicate balance between autoimmunity and immune tolerance. These drug-induced adverse events are termed immune-related adverse events (irAEs) (7). Trastuzumab is an antibody-drug conjugate (ADC) that exerts its anticancer effect by specifically delivering cytotoxic drugs to cancer cells. Following internalisation within cancer cells, ADCs release their highly active cytotoxic substances, thereby inducing cancer cell apoptosis (8, 9).

Adverse reactions to immune checkpoint inhibitors are characterised by an absence of specific clinical manifestations and the potential to trigger various inflammatory diseases, including cutaneous inflammation, colitis, gastritis, focal or diffuse pulmonary inflammation, glandular or organ attacks, and inflammatory arthritis (10, 11). A review of the literature reveals numerous case reports of PD-1 immune checkpoint inhibitor antibodies causing adrenal insufficiency. For instance, Yukino Katakura et al. published a case in Frontiers in Endocrinology describing a patient with malignant melanoma treated with nivolumab and/or ipilimumab. Following a period of approximately nine months of therapy, the patient exhibited symptoms of IAD (12). Common adverse reactions associated with trastuzumab include nausea, decreased appetite, fatigue, anaemia, thrombocytopenia, neutropenia, leukopenia, and vomiting (13, 14). A comprehensive review of the extant literature, both domestic and international, has failed to identify any reports of trastuzumab-induced adrenal insufficiency-related adverse events. However, there are reports of trastuzumab causing severe hypoglycaemia events (15). Given the absence of hormone level testing in the case, the possibility of isolated ACTH cannot be excluded.

The fundamental distinction between this case and previously documented cases is predicated on the patient’s initial presentation of hypoglycaemia. Following a total gastrectomy for gastric adenocarcinoma, the patient was misdiagnosed with dumping syndrome. The dumping syndrome is classified into two phases, namely the early and late phases, based on the symptoms exhibited by the patient. These symptoms may occur in a simultaneous or individual manner (16). The symptoms of early dumping syndrome manifest within one hour of eating and include gastrointestinal symptoms, such as abdominal pain, bloating, nausea and diarrhoea, and vasomotor symptoms, including flushing, palpitations, sweating, tachycardia, hypotension, fatigue, a desire to lie down and, rarely, syncope (17, 18). The manifestation of symptoms associated with late dumping syndrome typically occurs within a time frame of 1 to 3 hours following the ingestion of food, primarily manifesting as hypoglycaemia, driven by the postprandial hyperinsulinemic response triggered by incretin hormones (19, 20). Patients diagnosed with dumping syndrome may experience a range of symptoms, including palpitations, cold sweats, fatigue, and abdominal discomfort following the ingestion of food (21). However, these symptoms were not observed in the patient under consideration. Emergency physicians diagnose gastric dumping syndrome using gastric emptying studies, even though rapid gastric emptying is a key mechanism. However, the diagnostic accuracy of this method is low. It has been demonstrated that the initial rapid emptying of the stomach may trigger dumping symptoms, such as nausea. However, these symptoms can also result in a delay in the emptying of the stomach, thereby ensuring that overall emptying rates remain within normal ranges. Presently, the Oral Glucose Tolerance Test (OGTT) is regarded as the optimal diagnostic modality for dumping syndrome. A positive result is indicated by the presence of hypoglycaemia in the late phase (120–180 minutes) or an increase in haematocrit exceeding 3% in the early phase (30 minutes) (22, 23). In instances where patients present with a pertinent surgical history and concomitant symptoms, it is incumbent upon physicians to undertake a comprehensive analysis and meticulous differential diagnosis. In this case, subsequent testing of relevant hormones confirmed an isolated ACTH deficiency. Following the initiation of prednisone replacement therapy, the patient exhibited significant improvement in symptoms and correction of hypoglycaemia.

This case serves as a cautionary example, highlighting the necessity for healthcare providers to exercise vigilance when administering nivolumab and trastuzumab to patients with a history of cancer. In such cases, relevant hormone tests should be conducted with particular attention to symptoms such as uncorrectable hypoglycaemia, loss of appetite, nausea, vomiting, or fatigue, as these may indicate underlying issues that require immediate attention. This vigilance is crucial to prevent missed or misdiagnoses that could delay appropriate treatment and worsen the patient’s condition.

## Data Availability

The original contributions presented in the study are included in the article/supplementary material. Further inquiries can be directed to the corresponding author.
